# Unlocking dormant Li^+^ pathways drives fast ion transport in Li_4_Ti_5_O_12_ oxide spinels

**DOI:** 10.1126/sciadv.aef5575

**Published:** 2026-08-19

**Authors:** Bernhard Gadermaier, H. Martin R. Wilkening

**Affiliations:** Institute of Chemistry and Technology of Materials, Graz University of Technology (NAWI Graz), Stremayrgasse 9, A-8010 Graz, Austria.

## Abstract

Li-rich lithium titanate (Li_4+*x*_Ti_5_O_12_, *x* > 0) is known for superior ionic conductivity, yet we show that stoichiometric Li_4_Ti_5_O_12_ (LTO, *x* = 0), typically characterized by sluggish ion dynamics, can be transformed into a fast ion conductor without changing its Li content. Local defects, most notably oxygen vacancies, introduced by vacuum treatment activate a previously inaccessible 8*a*-16*c*-8*a* diffusion pathway in stoichiometric LTO, markedly enhancing Li^+^ mobility throughout the bulk. Using a synergistic combination of impedance spectroscopy, solid-state nuclear magnetic resonance (NMR), and electron paramagnetic resonance (EPR), we resolve the diffusion processes responsible for this transformation. Lithium NMR unambiguously shows that a formerly localized Li^+^ hopping process becomes long-range transport after vacuum treatment, evidencing the activation of extended diffusion pathways. Atomic-scale insights reveal defect-driven structural and dynamical priming that enables rapid Li^+^ insertion, establishing zero-strain LTO as a leading anode for solid-state lithium batteries. Defect-mediated transport emerges as the key mechanism underlying these dynamics, resolving the long-standing conductivity puzzle of stoichiometric spinel LTO (*x* = 0) and indicating transferable pathways in related spinel-type ion conductors with similar Li^+^ distributions.

## INTRODUCTION

Electrochemical energy storage devices are important in achieving a fossil-independent economy (*1*). Apart from mobile and automotive (*2*, *3*) applications, such devices are also important for grid stabilization (*4*, *5*), especially considering the rising amount of intermittent renewable energy from wind, water, and sun (*1*). In the last decades (*6*), enormous improvements in Li-ion batteries have rendered them the most promising solution for this problem. These achievements rest on the detailed studies of active materials, especially concerning their electrochemical properties (*7*). Most importantly, detailed studies on their ionic and electronic conductivity revealed important routes for their optimization. The case of LiFePO_4_ (*8*, *9*), a material initially considered unsuitable in batteries but later found to be an extremely efficient active material, when nanosized to improve ionic conduction and carbon-coated to enhance electronic conduction (*10*), is just one example that highlights the importance of fundamental studies that propel battery performance in unexpected ways.

Lithium pentatitanate (Li_4_Ti_5_O_12_, LTO) is a well-characterized negative electrode active material used in commercial lithium-ion batteries that offers excellent structural stability, high-rate capability, and safety features (*11*, *12*). Its cubic spinel structure allows the reversible insertion of *x* = 3 Li^+^ ions (Li_4+*x*_Ti_5_O_12_) at approx. 1.55 V vs Li/Li^+^ (*13*) in octahedral voids without introducing strain. During Li-insertion and reduction of the Ti^4+^ centers, ionic and electronic conduction are improved (*14*). In its initial, pristine state (*x* = 0), it is, however, an inferior conductor both for electrons and ions and it remains puzzling how such a material shows such excellent performance in batteries.

Its rather flat voltage plateau during Li-insertion suggests that a phase conversion occurs and that it behaves as a typical two-phase material. In such a scenario, the active material particles would be converted starting from the surface, which becomes conductive and progresses into the core (*15*). While seemingly at odds with the electrochemical picture, detailed NMR studies point to LTO, with *x* slightly larger than 0, behaving instead as a solid solution rather than a two-phase conversion material (*16*, *17*). In this case, it seems surprising that the reaction kinetics of pristine LTO are as fast as they are, especially given its extremely low ionic and electronic conductivity for *x* = 0.

It is known that LTO experiences an enhancement of its performance in electrochemical cells if oxygen vacancies are introduced (*18*, *19*). To gain further insight into the dynamics in the unlithiated, pristine material, we performed a comprehensive approach consisting of multiple analytical techniques and found that oxygen vacancies in the material are an important facet that enhances the ionic conductivity significantly. Specifically, nuclear magnetic resonance (NMR) relaxation rate measurements gave insights into the Li-ion dynamics and revealed two bulk diffusion processes. Conductivity measurements allow us to correlate these dynamic processes with electrical properties, highlighting the influence of different annealing temperatures on conduction mechanisms.

Beyond their relevance for energy storage, these findings link LTO to ionotronics (*20*, *21*), where mobile ions, not electrons, define device functionality. Indeed, recent studies demonstrate resistive switching in LTO via field-induced phase separation and Li^+^ migration (*22*), potentially mediated by oxygen-vacancies, pointing to its potential as a memristive system. The oxygen-vacancy-enabled Li^+^ transport mirrors conduction in oxide-based memristors and underpins resistive switching and neuromorphic behavior (*23*).

## RESULTS AND DISCUSSION

### Conductivity measurements

**Fig. 1.**
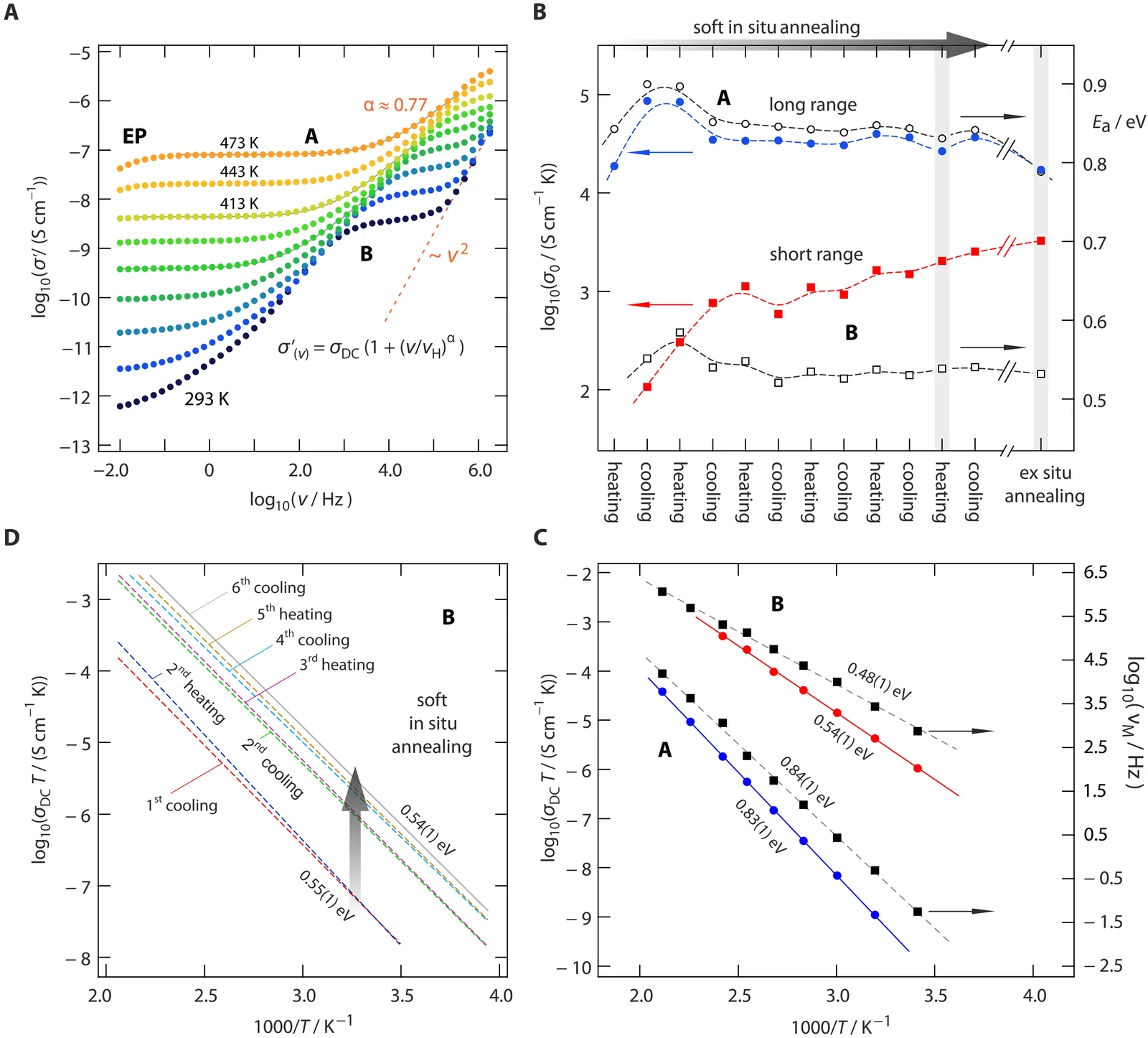
Conductivity behavior of LTO. (**A**) Conductivity spectra of in situ annealed LTO. (**B**) Arrhenius plot showing the effect of the in situ annealing on the ionic conductivity of the short-range process B. (**C**) During the in situ annealing, the factors defining the Arrhenius equation approach those of the ex situ annealed sample. The long-range process A remains largely unaffected. In contrast, the pre-exponential factor of the short-range process B increases with every cycle, approaching that of the ex situ annealed sample. This observation is consistent with an increase in the charge carrier density, on which the pre-exponential factor is known to depend. (**D**) Arrhenius plot of the two different conductivity processes (6th cycle) showing how the DC-conductivity and the dielectric relaxation frequencies, i.e., the peaks in *M*″, are affected by temperature.

During consecutive conductivity measurements in alternating current (AC) mode on Li_4_Ti_5_O_12_, conducted while cycling the temperature between 20°C and 200°C under a nitrogen (N_2_) atmosphere, we observed changes in the direct current (DC) conductivity (σ_DC_) of the sample, along with the emergence of a new conduction process (see Fig. 1A). In Fig. 1A the corresponding conductivity isotherms σ′_(ν)_ are shown. Initially, LTO was dried under a vacuum at 105°C to remove moisture, and a single bulk conduction plateau A (see fig. S1A) was observed. However, a second conductivity plateau (Plateau B in Fig. 1A), associated with a distinct bulk electrical process, emerged during subsequent heating and cooling cycles. This plateau B, located at higher frequencies, exhibited significantly higher conductivity, indicating a second conduction process within the grains due to highly mobile charges – hence a second bulk process is found (see below).

To analyze the full shape of the isotherms σ′_(ν)_, we used Jonscher’s power law formalism (*24*), fitting σ′_(ν)_ = σ_DC_ (1 + (ν/ν_H_)^α^), to extract DC conductivities σ_DC_, hopping rates ν_H_ and the exponents describing the slope of the conductivity (i) between the two plateaus and (ii) in the high-frequency regime. Electrode polarization effects are best seen in the isotherm recorded at 473 K; they help identifying the plateau A as caused by ions that are blocked by the gold electrodes. Notably, at ambient temperatures (293 K) the high-frequency regime shows a steep rise associated with a ν^2^-dependence. This quadratic frequency dependency identifies this process as a resonance phenomenon, possibly due to a local dipolar relaxation. In most ionic conductors, the high-frequency plateau is followed by the nearly constant loss regime, which is characterized by an exponent α = 1 or slightly lower.

The extracted DC conductivities σ_DC_ were analyzed in the frame of a classical Arrhenius relation σ_DC_*T* = σ_0_ exp(−*E*_a_/(*k*_B_*T*)) where σ_0_, *E*_a_, *k*_B_, are the pre-exponential factor that depends on the charge carrier density, the activation energy of conduction and Boltzmann’s constant, respectively. During successive heating and cooling cycles, we find that soft in situ annealing increases σ_DC_ of the short-range conduction process B by almost two orders of magnitude (see Fig. 1B). This change is best described by the pre-exponential factor σ_0_ and the activation energy *E*_a_ (see Fig. 1C). Both factors remain stable after the second cycle for the long-range process A. The short-range conduction process B, which only emerged after the first heating cycle, showed a continuously increasing pre-exponential factor σ_0_ while the activation energy *E*_a_ stabilized after a few cycles at 0.55 eV.

We have previously demonstrated that consecutive heating and cooling cycles should be performed to identify the stability and true nature of the conduction in ionic conductors (*25*). A stable activation energy indicates that the diffusion process remains unaltered. The increase of the pre-exponential factor σ_0_ shows that the fraction of ions diffusing via this process increases from cycle to cycle (see Supplementary Text S1 for discussion). We hypothesize that an in situ annealing process enables this local conduction process B. Hence, we annealed a sample at 300°C under vacuum for 60 hours to test this hypothesis. This ex situ annealed sample indeed shows the second plateau B already in the first heating cycle, and both the pre-exponential factors σ_0_ and activation energies *E*_a_ are very similar to those of the in situ annealed sample. Notably, during in situ annealing, both factors approach the values found also in the ex situ annealed sample (see Fig. 1C).

### Electric modulus analysis

To further characterize the two conduction processes in LTO, we analyzed the imaginary part of the electric modulus, *M*″, which emphasizes dielectric relaxation and offers deeper insight into ion dynamics in the spinel structure (see fig. S1B). Most notably, the *M*″ peaks enable a much clearer separation of distinct conduction processes and scale proportionally with the respective associated capacitances (*26*). Both electrical processes A and B show capacitances in the order of 25 pF and 5 pF, respectively, which is typical for bulk conduction processes.

The dielectric relaxation frequencies marked by peaks in *M*″ are thermally activated and follow an Arrhenius relation. The relaxation frequencies recorded during the last (6th) heating cycle are shown in Fig. 1D along with the corresponding conductivities. The conduction process A shows equal activation energies of 0.84 eV for both the conductivity and the relaxation frequencies. Typically, this is the case as the increase in the conductivity is based on an increased relaxation frequency, i.e., an increase in ionic hopping rate (*27*). Worth mentioning, while the conduction process seen in Plateau B (Fig. 1A) is activated by 0.54 eV, the electrical relaxation process is activated by only 0.48 eV. This exact activation energy was reported by Ren *et al.* (*28*) and Ziebarth *et al.* (*29*). The difference between these activation energies might be traced to a temperature-dependent fraction of ions diffusing via this process (*30*). The conductivity experiences an activation energy based on increasing hopping rates and an increasing number fraction of ions involved in this process. An increasing number of ions participating in this process could also explain the increase of the pre-exponential factor (see Supplementary Text S1 for further discussion). We hypothesize that conduction process B is associated with defect complexes of Li ions. As temperature increases, the degree of complex association decreases, resulting in an increase in charge carrier density; a detailed discussion follows below.

### Permittivity analysis

**Fig. 2.**
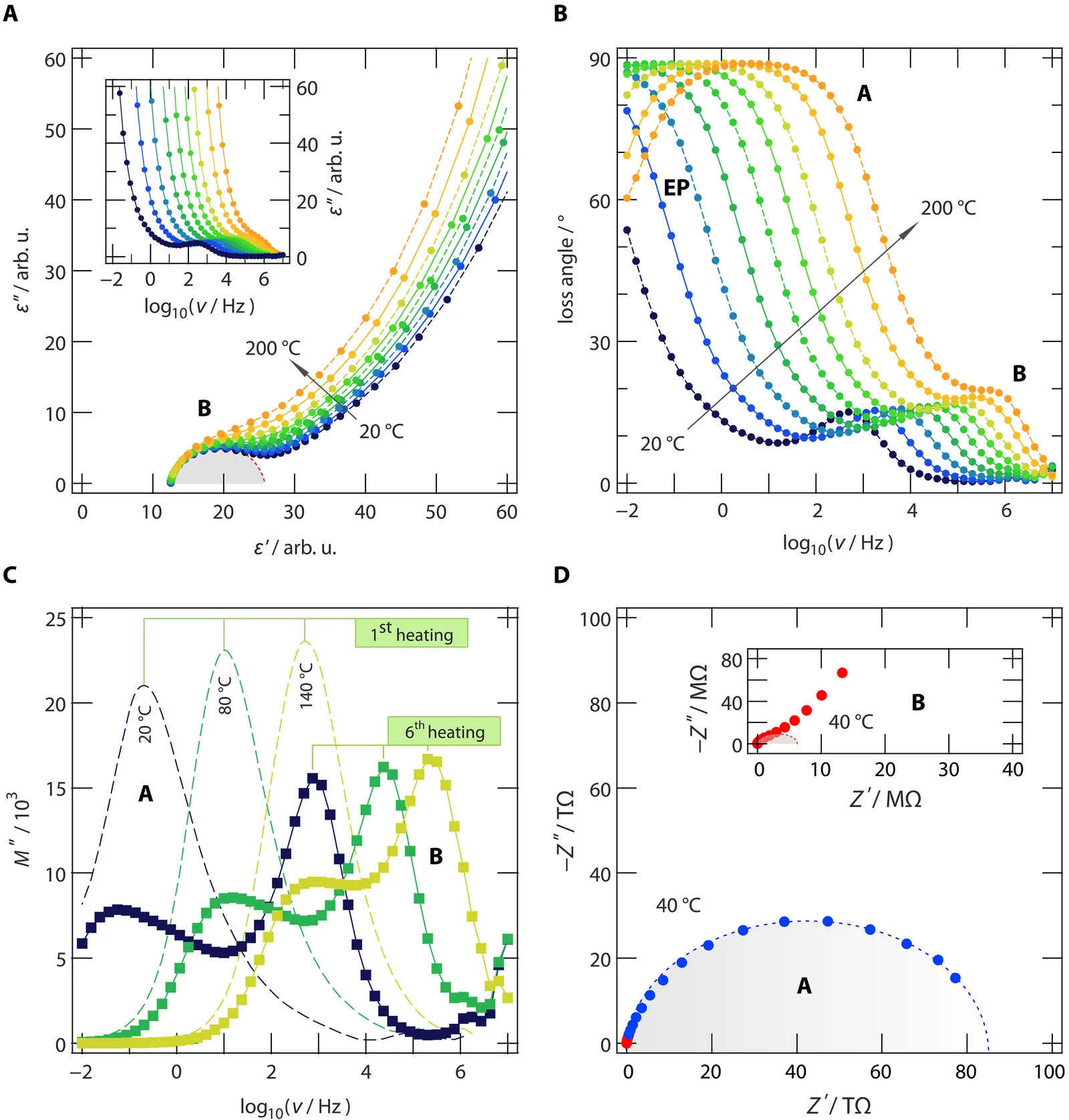
Electrical characterization of LTO, permittivity, loss angle, electric modulus and impedance. Different representations of impedance response of the LTO-105 sample recorded at temperatures between 20°C and 180°C during the 6th heating cycle. (**A**) The Argand plot shows the complex permittivity. A dielectric relaxation process, evident by the semicircle is well resolved at low-T but becomes increasingly masked at higher temperatures by the long-range diffusion process A. The inset shows the imaginary part of the permittivity as a function of the frequency. (**B**) The loss angle passes through a maximum at high frequencies (see peak B) and low frequencies (see peak A) as the impedance response becomes foremost dominated by resistive behavior, i.e., conduction. Note that at 180°C, the phase angle drops at low frequencies due to the ion-blocking effect of the Au-electrode (EP). (**C**) Spectroscopic plot of the imaginary part of the Modulus. This representation reveals the dielectric relaxation process in the form of peaks and highlights capacitive contributions, i.e., the peak height is approximately inversely proportional to the capacitance. Here, the very similar peak heights indicate that both processes are characterized by capacitances lying at least in the same order of magnitude. For comparison, the 1st heating cycle, which is characterized by only one process, is also shown. (**D**) Complex plane plot of the impedance recorded at 40°C showing the highly resistive transport long-range process A and less resistive short-range conduction process B, see inset; note the different scaling of the axis.

To analyze the impedance data in more detail, we examined the complex permittivity, ε* = ε′ + *i*ε″, which characterizes how the material responds to alternating electric fields. In simpler terms, permittivity reflects how well the material can store and dissipate electrical energy, revealing subtle features of ion movement that are not always evident in impedance plots alone (see Fig. 2). For a spatially localized short-range diffusion process, a Debye-like relaxation process is expected. This can be thought of as ions hopping forth and back in a confined region, producing a characteristic “relaxation” signature. Mathematically, the complex permittivity in such a case follows ε* = ε_∞_ + Δε/(1 + *i*ωτ_D_), where ε_∞_ is the high-frequency limit of the permittivity, Δε is the relaxation strength, ω is the angular frequency, and τ_D_ is the characteristic relaxation time. When plotted as the imaginary part versus the real part, this relaxation appears as a perfect semicircle, similar to the well-known Nyquist semicircle in impedance spectroscopy. Indeed, at low temperatures, we observe a semicircle (process B in Fig. 2A) indicating a localized short-range Li^+^ diffusion mechanism.

With increasing temperatures, the semicircle becomes less pronounced and the minimum in the imaginary part of the permittivity, ε″, disappears (see also inset in Fig. 2A). This is because the long-range conduction process A shows a much higher activation energy of 0.84 eV, hence its contribution to the imaginary part of the permittivity ε″ ∝ σ_DC_ increases and finally masks the dielectric relaxation process in this representation. In other words, ions that initially hop in a confined region (process B) eventually contribute to long-range transport (process A) over longer timescales and lower frequencies. Since these two conduction processes are physically linked, their permittivity contributions overlap and cannot simply be separated (*31*). At even higher frequencies, ε″ increases again with a linear dependence on the frequency, and hence the conductivity shows a quadratic dependency on the frequency (see Fig. 1A). Both conduction processes are also clearly marked by maxima in the loss angle while the electrode polarization (EP) is evidenced by the decrease of the loss angle at high temperatures and low frequencies (see Fig. 2B). The presence of electrode polarization confirms that the observed conduction process is indeed ionic.

### Electric relaxation at higher temperatures and the effect of heating cycles

To unmask the relaxation processes at higher temperatures, we analyzed the electric modulus, which is the inverse of the complex permittivity. The imaginary part of the electric modulus, *M*″, is particularly suited to reveal dielectric relaxation processes even in the presence of conduction, which occludes these processes in the permittivity plots. It highlights the conduction processes as maxima at specific relaxation frequencies ν_M_. Note that during the first heating cycle, only the conduction process A is visible whereas the 6th heating cycle shows the fast conduction process B well-pronounced (see Fig. 2C). Finally, the Nyquist plot showing the impedance data recorded at 40°C highlights a slightly depressed semi-circle for the conduction process A and a hardly resolved semi-circle for the faster conduction process B (see inset in Fig. 2D). Hence, from our impedance spectroscopy analysis we can deduce that during consecutive heating and cooling cycles as well as during ex situ annealing of LTO a second bulk conduction process appears. While the slower process A is unaffected by thermal treatments, the short-range conduction process B is affected and shows a consecutively increasing pre-exponential factor that is approaching that of the ex situ annealed sample (see Fig. 1A). Furthermore, at high frequencies, a resonance absorption is observed in the conductivity isotherms and is manifests itself by the quadratic frequency dependency of σ′_ν_. To gain insight into the effects of thermal treatment, we used Fourier-transform infrared (FTIR), electron paramagnetic resonance (EPR), and high-resolution NMR spectroscopy.

**Fig. 3.**
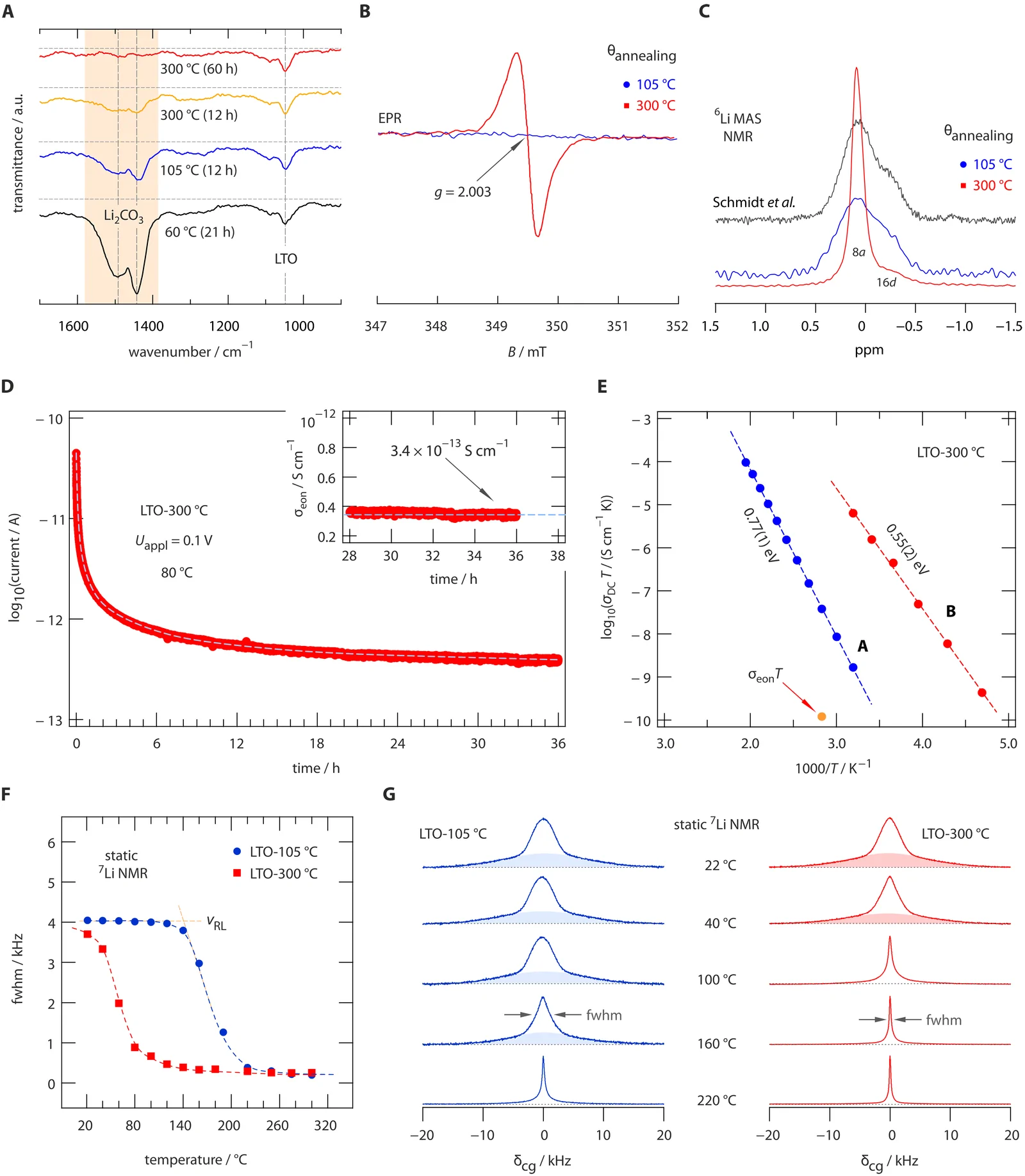
Surface properties, electronic conductivity, insights from EPR and NMR. (**A**) FT-IR spectra of LTO previously dried under different conditions reveal how the Li_2_CO_3_ surface layer progressively diminishes. (**B**) Electron paramagnetic resonance lines reveal the presence of unpaired electrons in the sample annealed for 60 h at 300°C in vacuum. No indication for Ti^3+^ (*g* = 1.965) was found at room temperature and 180 K. (**C**) ^6^Li MAS NMR spectra were recorded at an MAS frequency of 25 kHz. While the sample annealed at 105°C shows a very broad line that perfectly matches that recorded by Schmidt *et al.* (*17*), the 300°C-sample shows a very sharp line (8*a*) and a broad component (16*d*). (**D**) Potentiostatic polarization curve recorded at 80°C, applying a voltage difference of 100 mV to an LTO-300 pellet contacted by ion-blocking gold electrodes. The inset shows an enlarged view of the long-time polarization region yielding the electronic conductivity σ_eon_. (**E**) Arrhenius plot showing the ionic conductivity of the short-range and long-range process and the electronic conductivity σ_eon_ at 80°C. (**F**) ^7^Li central linewidths of the pristine (LTO-105°C) and annealed sample (LTO-300°C) as a function of temperature. See text for further discussion. (**G**) Selected ^7^Li spectra of the pristine and annealed sample were recorded at the indicated temperatures. With increasing temperature, the central line narrows and the quadrupolar signal (colored area) narrows and loses intensity.

Laboratory x-ray powder diffraction was used to analyze structural details; it indicates highly crystalline LTO without side phases (see fig. S1C). The method is, however, not sensitive enough to characterize minute changes in the sample such as a low number of low-dimensional defects (point defects including vacancies). Water vapor and CO_2_ in the atmosphere can lead to the formation of Li_2_CO_3_ on the surface of lithium-rich particles (*32*). Due to the rather poor ionic conductivity of Li_2_CO_3_ such a layer could pose a high resistance to the intragrain conduction. It would cause a significant accumulation of charges at the interfaces, i.e., at the grain boundary regions. No such effect was, however, observed in impedance spectroscopy. Nevertheless, FTIR spectroscopy was conducted on annealed samples to understand how thermal treatment affects them. The IR spectra indicate the presence of Li_2_CO_3_, which can be removed by thermal treatment (see Fig. 3A) and the lack of any Li_2_O suggests that it is re-incorporated into LTO. After removing Li_2_CO_3_ as in the LTO-300 sample, the conductivity remained unaltered (see Fig. 1C), which underpins our assignment of the conduction processes to bulk processes.

### Paramagnetic centers through oxygen loss and EPR analysis

Since thermal treatment enabled a local diffusion pathway within the bulk regions of the material, we suspected that oxygen deficiency due to annealing under extremely low oxygen partial pressures, such as in a vacuum during ex situ annealing or in freshly evaporated N_2_ during in situ annealing, might be the key factor. Oxygen removal from the sample is a formal reduction as two electrons remain within the sample. In the Kröger-Vink notation (*33*), this process $O_{O}^{x}\to 1/2O_{2}+2e^{\prime }+V_{O}^{••}$ would generate two electrons and a positively charged oxygen-vacancy. The electrons can be trapped in an oxygen vacancy (*34*) $V_{O}^{••}+e^{\prime }\to V_{O}^{•}$, or reduce a transition metal ${\text{Ti}}_{\text{Ti}}^{x}+e^{\prime }\to {\text{Ti}}_{\text{Ti}}^{\prime }$ which would generate paramagnetic centers with a distinct signature in EPR spectroscopy. EPR spectroscopy confirmed the presence of paramagnetic centers with a Landé factor, *g*, of 2.003, indicative of unpaired free electrons (*35*) (see Fig. 3B). Paramagnetic Ti^3+^ centers, ${\text{Ti}}_{\text{Ti}}^{\prime }$, are expected for reasons of charge compensation but were not detected. Even at 180 K, no resonance corresponding to Ti^3+^ (*g* = 1.96) (*36*) was observed. Cooling the sample to lower temperatures would likely increase the sensitivity and reveal the presence of paramagnetic Ti^3+^ centers (*37*). To study the effect of oxygen vacancies, which contain unpaired electrons, on ion dynamics, we used NMR to gain complementary insights.

### NMR as a direct tool to probe rapid ion dynamics in annealed LTO

We used ^6^Li magic angle spinning (MAS) NMR to analyze both samples in more detail while focusing on the mobile cation. The ^6^Li nucleus is a spin-1 nucleus and thus much less sensitive to paramagnetic centers that could mimic the signature of fast ionic motion or lead to line broadening, obscuring the insight into local structures (see Fig. 3C). The spectra revealed a much sharper line in the ex situ annealed sample compared to the pristine sample, which points to increased Li-ion dynamics in the annealed sample. The recycle delay between each MAS NMR line measurement, which was necessary to acquire quantitative spectra, gives at least a qualitative insight into the diffusivity of ^6^Li.

In the pristine sample, a recycle delay of 600 s was required to obtain a quantitative ^6^Li MAS NMR spectrum. In contrast, for the sample annealed at 300°C, much faster longitudinal relaxation rates allowed for significantly shorter delays. For comparison, a delay time of just 100 s yielded a spectrum with an 8*a*:16*d* intensity ratio of 78:22, which reflects the structural population ratio. For comparison, a third ^6^Li spectrum recorded using 440 s delay times is also shown in Fig. 3C. Importantly, a short recycle delay of only 5 s was sufficient to already detect an extremely narrow line (0.13 ppm) corresponding to Li on 8*a*. This pronounced line narrowing is consistent with rapid Li^+^ exchange processes in the annealed sample.

Site-specific relaxation rate measurements at room temperature under MAS conditions showed that, for the annealed sample, the *T*_1_ time of ^6^Li on 8*a* is only 19 s, whereas ^6^Li on 16*d* exhibits a significantly longer *T*_1_ time of 137 s (see fig. S2). Annealing evidently enhances NMR spin-lattice relaxation for ^6^Li. While paramagnetic centers may contribute to the shortening of *T*_1_ times to some extent, the motionally narrowed 8*a* ^6^Li NMR signal indicates that rapid Li^+^ diffusivity between 8*a* and 16*c* sites, triggered by oxygen vacancies, is the primary cause of the accelerated longitudinal relaxation (*38*).

For comparison, similarly narrow ^6^Li MAS NMR lines (0.11 ppm) are obtained in slightly chemically lithiated LTO, showing rapid ion exchange processes as well (*17*). Recently, Zhang *et al.* (*14*) proposed that fast Li ion diffusion is enabled by distorted Li polyhedra generated during Li insertion. Although no lithium is inserted in our case, the Li polyhedra are distorted due to oxygen vacancies, and a similarly enhanced diffusivity is observed. The narrow 8*a* ^6^Li MAS NMR line detected in this study closely resembles that of slightly lithiated LTO (*17*). Hence, modifying the defect structure, either by inserting small amounts of additional Li^+^ or by altering it through annealing, activates the same fast Li^+^ diffusion process, transforming poorly conducting LTO into a fast ion conductor.

### Electronic conductivities

The sample annealed at 300°C showed an EPR signal indicative of a paramagnetic species (see Fig. 3B), which may introduce electronic conductivity. Therefore, we performed a polarization measurement to estimate the electronic conductivity. For consistency, the pellet was loaded into a sample holder that allowed impedance and polarization measurements without manipulating the sample. We first performed impedance measurements between 240°C and −60°C (see Fig. 3E). The sample was polarized for 36 hours at 80°C and a final impedance measurement verified the integrity of the sample. The polarization measurement indicates that the electronic conductivity is at least lower than 10^−12^ S cm^−1^ (see Fig. 3D). The ionic conductivity of the long-range process A is, at 80°C, at least two orders of magnitude higher (see Fig. 3E).

### Variable-temperature ^7^Li NMR line shape measurements

Before we analyze the ^7^Li relaxation rates in detail, we will examine the static ^7^Li lines obtained at different temperatures for the pristine (LTO-150) and annealed (LTO-300) samples (see Fig. 3, F and G). The ^7^Li NMR signal of LTO-150 (spin quantum number *I* = 3/2) comprises a narrow central line corresponding to Li on 8*a* and 16*d*, along with a considerably broader quadrupolar component (*16*) caused by the interaction of the quadrupolar moments with non-vanishing electric field gradients. In the absence of ^7^Li motion, i.e., at low temperatures, dipolar interactions determine the ^7^Li NMR rigid lattice linewidth (ν_RL_) of the central line. With increasing temperature, the jump rate increases, and, when it is in the order of ν_RL_, the line narrows. Inhomogeneities of the magnetic field limit the narrowing to a finite line width (fwhm, full width at half maximum). The pristine sample, LTO-105, shows an onset of line narrowing at about 140°C, at this temperature, the Li jump rate is in the order of ν_RL_, i.e. 4 kHz. In contrast, the rigid lattice linewidth of the annealed sample, LTO-300, is not observed even at room temperature. The Li 8*a* jump rate is at least already in the order of 4 kHz and, hence, much higher in the annealed sample than in the pristine one. Selected ^7^Li NMR lines are shown in Fig. 3G and comprise an overall central line on top of the quadrupolar signals (colored region in Fig. 3G). As the temperature increases, a significant narrowing of the central line is observed, primarily due to motional averaging of dipolar couplings. This effect is largely driven by 8*a*-16*c* jumps. In contrast, the less mobile Li ions occupying 16*d* sites contribute to the low-intensity baseline of the narrowed ^7^Li NMR central line. At sufficiently high temperatures, however, these 16*d* Li ions also begin to participate in the motional averaging, further contributing to the line narrowing. At even higher temperatures also the quadrupolar signal is affected by motional averaging and finally vanishes with increasing temperature (see also Schmidt *et al.* (*16*)). These pronounced differences in ^7^Li NMR line narrowing of the central intensities are fully consistent with the ^6^Li MAS NMR results and indicate increased Li^+^ jump rates involving the 8*a* sites in the annealed, oxygen-deficient sample.

### Diffusion-induced NMR rate peaks

**Fig. 4.**
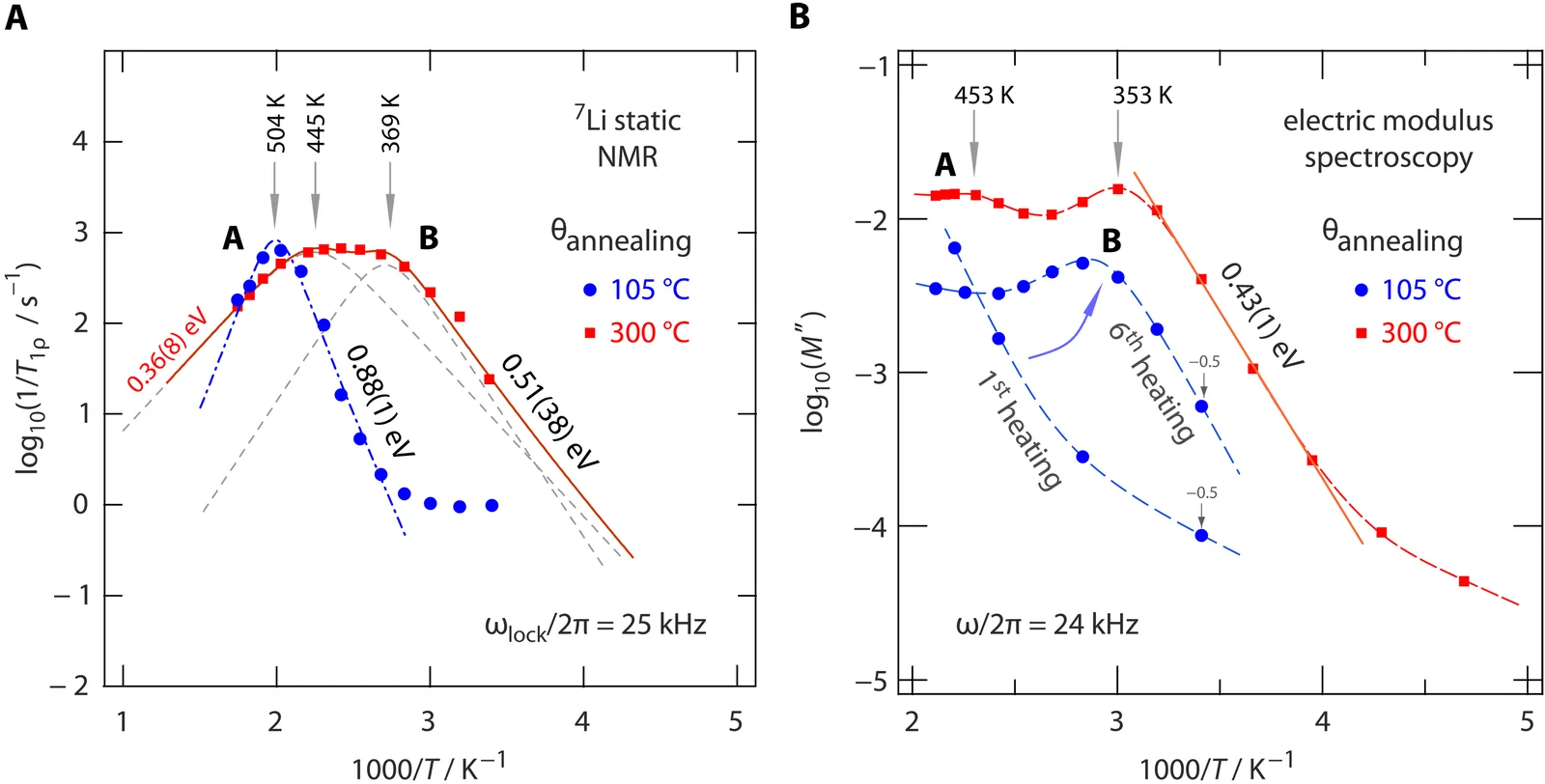
Variable-temperature NMR and electric modulus measurements. (**A**) ^7^Li spin-lattice relaxation rates recorded in the rotating frame of reference (ν_lock_ ≈ 25 kHz) showing the presence of faster diffusion processes in the oxide-deficient sample (annealed at 300°C). (**B**) Variable-temperature isofrequency (ν = 24 kHz) modulus analysis. During in situ annealing, a peak located at lower Temperatures appears which fits well to that observed in the sample annealed at 300°C. The peaks indicate that rather fast dielectric relaxation processes appear.

To gain further insight into the Li-ion dynamics, both the pristine sample and the ex situ annealed sample (300°C for 60 hours) were analyzed using temperature-dependent ^7^Li NMR relaxation rate measurements *R*_1ρ_(1/*T*) in the rotating frame of reference (see Fig. 4A). These measurements probe the spectral density function of the typically exponentially decaying autocorrelation function (*16*, *39*) of the ionic motion at a chosen locking frequency ω_lock_/2π to extract specific correlation times, and activation energies. The fitting routine to extract the relaxation rates is discussed in the Supplementary Materials, see fig. S3. These relaxation rates *R*_1ρ_ show a maximum when the ionic jump rate equals the applied angular locking frequency ω_lock_ (see Supplementary Text S2 for a detailed discussion). The jump rates are thermally activated following an Arrhenius relation, which relates the peak’s slopes to the activation energies, i.e. energy barriers the ion perceives during diffusion. Diffusion-induced rate peaks, hence, give a unique insight into a diffusion process.

The pristine sample, LTO-105, shows a single, symmetric, diffusion-induced rate peak at 504 K superimposing a non-diffusive background. The latter dominates the rates below 100°C and is integrated in our peak-fitting routine. The ex situ annealed sample LTO-300 reveals two diffusion-induced rate peaks at 369 K and 445 K. While the latter is likely the same accelerated diffusion process also seen in the pristine sample, the second NMR rate peak at lower temperatures indicates that a new diffusion process emerged during annealing. This process, primarily driven by 8*a* Li ions responsible for the narrow line in ^6^Li MAS NMR, suggests jump rates on the order of the locking frequency (25 kHz) already at 369 K, thereby indicating a significantly faster diffusion mechanism. This finding is similar to what we observed in our impedance measurements. The pristine sample shows only one conduction process A but a second one, B, emerges during in situ annealing and matches the situation in the ex situ annealed sample.

### Activation energies from NMR

We will discuss the rate peaks in more detail before we compare these findings with the impedance data. In the pristine sample, this symmetric, diffusion-induced NMR relaxation rate peak at 504 K shows an activation energy of 0.88 eV (Fig. 4A). By measuring the ^7^Li NMR spin-alignment echo in LTO, which provides microscopic access to long-range Li-ion transport, Wilkening *et al.* determined an activation energy of 0.86 eV for LTO (*40*). Previously measured ^7^Li *R*_1ρ_ rates at different locking frequencies ω_lock_ and different magnetic field strengths, i.e. angular Larmor frequency ω_L_, show activation energies spanning from 0.55 eV to 0.74 eV (see Table 1). For example, Schmidt *et al.* (*16*, *41*) obtained 0.62 eV and 0.58 eV, whereas Wilkening *et al.* (*40*) reported 0.74 eV and Hain *et al.* (*42*) presented a value of 0.55 eV (see Table 1).

**Table 1.** NMR activation energies. Activation energies were deduced from ^7^Li NMR spin-lock relaxation rates measured at different Larmor and locking frequencies; the variability in values may also be attributed to sample-to-sample differences.

	ω_lock_/2π	ω_L_/2π	*E*_a_/eV
Schmidt *et al*. (*16*)	20 kHz	194 MHz	0.62
Schmidt *et al.* (*41*)	20 kHz	116 MHz	0.58
Wilkening *et al.* (*40*)	10 kHz	78 MHz	0.74
Hain *et al.* (*42*)	13.1 kHz	116 MHz	0.55
This study (process A)	25 kHz	116 MHz	0.88
This study (process B)	25 kHz	116 MHz	0.51

The differences in the activation energies might be related to the exact measurement conditions; most likely, minor impurities and sample treatment variations influence the observed activation energies. Exhaustive impedance measurements by Fehr *et al.* (*43*) already suggested, that Li^+^ diffusion in LTO depends to a much larger degree on the thermal history of the sample and atmospheric conditions (see also Fig. 1C). Alternatively, as already pointed out previously by some of us (*40*), the motional autocorrelation function might be of a stretched exponential nature. In this case, the activation energies extracted from the low-*T* flank become frequency dependent, yielding systematically lower values at higher probing frequencies (*44*).

The ex situ annealed sample shows two diffusion-induced ^7^Li NMR relaxation rate peaks appearing at 369 K (A) and 445 K (B) (see Fig. 4A). NMR peak A is also observed in the pristine sample and is presumably caused by the same diffusion process. The shift of the peak to lower temperatures indicates increased Li^+^ diffusivity. Peak B observed at 369 K is attributed to a new diffusion process with even higher jump rates that emerged during annealing. While these two peaks are not fully resolved, we used two symmetric rate peaks to fit the data and extract activation energies (see fig. S1 for details). Here, the new diffusion process B, inducing the peak at lower temperatures, shows an activation energy of 0.51 eV. Peak A at higher temperatures is characterized by a reduced activation energy of only 0.36 eV compared to its pendant in the pristine sample (see Fig. 4A). The ex situ annealing, mimicking the in situ annealing observed during the impedance measurements, enables a fast diffusion process resulting in a relaxation rate peak at temperatures as low as 369 K. Interestingly, Graf *et al.* found fast local diffusion processes (*45*). Thus, NMR detects two different diffusion-induced maxima only after annealing, suggesting that the two plateaus seen in conductivity measurements might be corresponding and are indeed based on ionic diffusion within the bulk structure.

### Comparing results from NMR with electric modulus data

To further compare our NMR data to the impedance data, we constructed isofrequency plots of the imaginary part of the modulus at a frequency similar to the locking frequency used in NMR (see Fig. 4B) (*46*). In these measurements, the imaginary part of the modulus is observed at a fixed frequency while the temperature is changed. For a Debye-like process, the imaginary part of the modulus follows *M*″ ∝ ωτ_D_/(1 + (ωτ_D_)^2^ whereas the characteristic relaxation time is thermally activated: τ_D_ = τ_D,0_ exp(*E*_a_/(*k*_B_*T*). This representation of the data typically shows a peak at the temperature at which ωτ_D_ ≈ 1 holds. The slopes of the peak give access to activation energies. The inverse of the characteristic frequency is assumed to be equal to the (mean) jump-rate of the ions τ_D_^−1^ = ν_jump_. While this type of analysis seems similar to the analysis of NMR relaxation rates, we have to stress that these measurements probe ion dynamics on a different physical basis. During the first heating cycle of the pristine LTO-sample, the data shows only a low-*T* flank as the peak was not accessible in the limited temperature range (see Fig. 4B). During the 6th heating cycle, the evolved diffusion process shows a peak at approximately 373 K indicating that the jump rate of the ions, diffusing via this pathway, is in the order of the measurement frequency. In the annealed sample, this process is visible from the beginning of the measurements and shows two peaks that partly overlap. The peaks are located around 453 K and 353 K which resembles the peaks seen in the NMR *R*_1ρ_ rates. The low-*T* flank of the *M*″ rate peak shows an activation energy of 0.43 (*1*) eV which is similar to the one observed by NMR, 0.5 (*4*) eV, and that observed for the relaxation frequency, 0.48 (*1*) eV (see Fig. 1D). Most importantly, both NMR and impedance spectroscopy show that a second diffusion process emerges as the sample is annealed in a slightly reducing atmosphere. Albeit the respective activation energies deviate slightly, they are well within reasonable ranges.

### Diffusion coefficients from NMR and comparison with conductivity data

The ^7^Li NMR rate peak gives direct access to a ^7^Li correlation time, which, within experimental errors, is equal to the inverse of the ^7^Li jump rate: τ_c_^−1^ ≈ ν_jump_ ≈ 2(2π ν_lock_). Assuming a jump distance of 1.81 Å (the distance between 16*c* and 8*a* sites in the crystal structure) and isotropic 3-dimensional diffusion, the NMR peak points to a diffusion coefficient *D*_NMR_ of 1.7 × 10^−15^ m^2^ s^−1^ at 504 K. This result can be converted into a conductivity value at that temperature and compared to the experimentally probed conductivity (see Supplemetary Text S3 for further details). The calculated conductivity of σ_NMR_, (0.9 to 1.2) × 10^−6^ S cm^−1^, is close to the measured long-range conductivity σ_DC_ of 0.25 × 10^−6^ S cm^−1^. We conclude that the same process is observed by both methods based on the similar activation energies measured by NMR (0.88 eV) and impedance (0.83 eV), and the agreement of calculated σ_NMR_ and extrapolated σ_DC_ conductivities. Hence, the long-range process A (see Fig. 1A) associated with a capacitance of 25 pF is a bulk process and not affected by the typical grain boundary contribution. We have demonstrated that the long-range conduction process A corresponds to the diffusion-induced NMR rate peak observed at elevated temperatures. The same comparison was performed for the NMR rate peak B appearing at lower temperatures. σ_NMR_ of (1.2 to 1.6) × 10^−6^ S cm^−1^ at 369 K is very similar to σ_DC_ of 0.5 × 10^−6^ S cm^−1^ measured at 373 K for the ex situ annealed sample. Measured and calculated conductivities based on NMR data differ only by a factor of 4, which is, given the assumptions, an excellent agreement. These findings strongly indicate that the conduction processes seen in impedance spectroscopy are indeed two bulk processes.

### Manipulation Li^+^ ion dynamics through annealing

Conclusively, LTO shows two bulk ionic conductivity processes, both seen by impedance and NMR measurements. The generation of oxygen vacancies enables faster Li^+^ diffusion. It is quite intuitive that oxygen vacancies impact Li^+^ self-diffusion in the pentatitanate, given its spinel structure. Oxygen vacancies cause the crystal lattice to, locally relax, resulting in slight distortions in the surrounding area. The multiple possible jump processes of Li-ions in LTO involve passing through a triangle of oxygen atoms within connecting polyhedral (*47*). Oxygen vacancies lower the energy barrier for these jumps, facilitating much faster diffusion, likely localized around the defect (*38*). Previous calculations by Heenen *et al.* (*48*) have shown that the self-diffusion of Li^+^ is significantly enhanced near defects in LTO. Recently, several studies pointed to enhanced conduction at the interface (*14*) of spinodal phases (*49*). Finally, overall improved battery performance of LTO has been associated with the formation of oxygen vacancies (*50*), which we assign to the formation of a second, fast bulk diffusion process. Likely, this process is also present in cycled LTO (*51*) and plays a critical role in its outstanding rate capabilities.

In summary, Li^+^ diffusion in pristine Li_4_Ti_5_O_12_ is strongly shaped by local structure and defect chemistry. We show that dynamic bulk properties, previously overlooked, play a critical role in enabling ion transport in this otherwise poor conductor. Impedance spectroscopy under very low oxygen partial pressure, combined with vacuum annealing, reveals two distinct conduction processes. While annealing removes surface Li_2_CO_3_, it has minimal effect on overall conductivity, indicating that bulk oxygen vacancies are the dominant contributors to enhanced Li + mobility. This enhancement is directly confirmed by ^6^Li MAS NMR and variable-temperature ^7^Li NMR relaxation displaying diffusion-induced maxima that map onto the impedance-derived processes. The faster diffusion process likely persists in electrochemically cycled Li_4+*x*_Ti_5_O_12_ (*x* > 0), providing a mechanistic basis for the high rate performance of LTO despite its low long-range conductivity at *x* = 0. Together, these results identify defect-mediated transport as the key mechanism enabling rapid Li^+^ hopping in zero-strain LTO and offer a general route to control ion dynamics in spinel-type conductors.

## MATERIALS AND METHODS

### Synthesis and characterization by x-ray

Li_4_Ti_5_O_12_ was obtained from Süd Chemie (EXM1037) in high purity and dried in vacuum (10^−6^ bar) at 60°C for 21 hours, 105°C for 12 hours, or 300°C for 12 hours or 60 hours. The crystal structure and purity of the sample were verified by x-ray diffraction using a Rigaku Miniflex 600 diffractometer that operates with Bragg Brentano geometry and uses CuK_α_ radiation. The powder diffractogram was recorded with a step size of 0.01° (10°/min) in the 2θ-range 10° to 90°. Rietveld refinement was carried out by using X’PertHighScorePlus (Panalytical).

### Electrical measurements

To analyze complex conductivities, the powder was uniaxially cold pressed into pellets (5 mm in diameter, 0.5 to 1 mm in thickness). After applying 100 nm thick Au electrodes employing sputtering, a Novocontrol Concept 80 broadband analyzer was employed to record conductivity isotherms; the frequency was varied from 10 mHz to 10 MHz. We measured impedance data at temperatures from 20°C to 200°C, cycling between these temperatures for 5 times. To exclude the influence of moisture, the sample was heated up to 180°C and cooled down to 20°C before isotherms were measured. In our setup, the temperature was automatically controlled employing a QUATRO cryosystem (Novocontrol), which uses a heating element to build up a specific pressure in a liquid nitrogen Dewar to create a constant flow of freshly evaporated N_2_ gas.

To estimate the electronic conductivity in the LTO-300 sample, we performed a potentiostatic polarization at a low voltage for 36 hours. We used the same setup as for the impedance measurements but used a passive impedance cell in combination with a ZG4 interface for accurate impedance spectroscopy before and after performing the polarization experiment. This particular setup enables switching between the measurements without manipulating the sample. The ZG4 interface is disconnected, and a Low-Current Interface (LCI) combined with a VersaSTAT3 (both from Princeton Applied Research) is connected to the sample cell. For temperature control, the same setup as for the impedance measurements was used.

### ^6^Li high-resolution NMR and IR

^6^Li Magic angle spinning (MAS) NMR measurements were performed on a Bruker AVANCE III spectrometer in a nominal magnetic field of 11.7 T. The samples were loaded into MAS rotors (ZrO_2_, 2.5 mm) under argon and spun at 25 kHz in a Bruker MAS probe head. ^6^Li Free induction decays (FID) were acquired at room temperature and the corresponding spectra were obtained via Fourier transformation. FIDs for the spectra were acquired using a single 90° excitation pulse followed by the FID acquisition. Using recycling delays of up to 10 min we collected up to 512 scans for a single spectrum. Infrared spectra of samples dried in a vacuum at different temperatures for different durations were obtained by an FTIR spectrometer (Bruker) – 10 scans were accumulated.

### ^7^Li relaxation rates in the laboratory and rotating frame of reference

We used a Bruker 300 MHz NMR spectrometer and a Bruker broadband probe to record ^7^Li relaxation rates in the laboratory frame (1/*T*_1_) and the rotating frame of reference (1/*T*_1ρ_) as well as ^7^Li NMR spectra. A saturation recovery pulse sequence with variable delays, *t*_d_, was used to record 1/*T*_1_ rates and is reported elsewhere (*52*, *53*). The magnetization transients *M*_z_(*t*_d_) were parameterized with stretched exponentials to extract the rate 1/*T*_1_ at Larmor frequencies of 116 MHz (^7^Li). These rates were recorded only at a few temperatures to calculate the relaxation rate and ensure that sufficient time (> 5*T*_1_) passed between each *T*_1ρ_ measurement.

The corresponding NMR relaxation rates in the rotating frame of reference 1/*T*_1ρ_ were acquired using a two-pulse spin-lock technique at a spin-lock frequency of 25 kHz (*52*–*55*). The first pulse flips the longitudinal magnetization by 90° where it is locked by the second pulse for a varying duration *t*_lock_. During these times, spin relaxation proceeds in the presence of a weak magnetic field oscillating in the kHz range, and the FID is recorded after the locking pulse. The magnetization transients follow a stretched exponential decay from which the diffusion-induced rate 1/*T*_1ρ_ was extracted. Typically, 4 scans were recorded and the FID obtained using the shortest time *t*_lock_, was Fourier transformed to obtain ^7^Li NMR spectra.

### EPR measurements

Continuous wave-electron paramagnetic resonance (CW-EPR) measurements were performed with a Bruker EMX X-band EPR spectrometer (100 kHz field modulation) equipped with a variable-temperature unit (Eurotherm B-VT 2000). EPR spectra were acquired at RT and 180 K using 2 mW microwave power and 0.1 mT field modulation. The *g* factor was calculated using 2,2-diphenyl-1-picrylhydrazyl (DPPH; *g* = 2.0036) as a reference.

## Supplementary Materials

### The PDF file includes:

Supplementary Text S1 to S3 Figs. S1 to S3 Legend for data S1 References
