# Supplementary material for: Unlocking dormant Li+ pathways drives fast ion transport in Li4Ti5O12 oxide spinels
Source: Sci Adv. 2026 Aug 19;12(34):eaef5575. doi: 10.1126/sciadv.aef5575 (PMC13488883; doi:10.1126/sciadv.aef5575)
Supplement: Supplementary file 1 [file sciadv.aef5575_sm.pdf]

Science Advances
AAAS

Supplementary Materials for

Unlocking dormant Li^+ pathways drives fast ion transport in $Li_4Ti_5O_{12}$ oxide spinels

Bernhard Gadermaier and H. Martin R. Wilkening

Corresponding author: Bernhard Gadermaier, bernhard.gadermaier@tugraz.at;
H. Martin R. Wilkening, wilkening@tugraz.at

Sci. Adv. **12**, eaef5575 (2026)
DOI: 10.1126/sciadv.aef5575

The PDF file includes:

Supplementary Text S1 to S3
Figs. S1 to S3
Legend for data S1
References

Other Supplementary Material for this manuscript includes the following:

Data S1

Results

Conductivity isotherm. Conductivity isotherms recorded during the 1st heating cycle indicated a quad-ratic frequency dependency of the real part of the conductivity but showed only a single conduction process (see fig. S1a). During the 6th heating cycle, two processes are clearly seen in the imaginary part of the modulus (see fig. S1b).

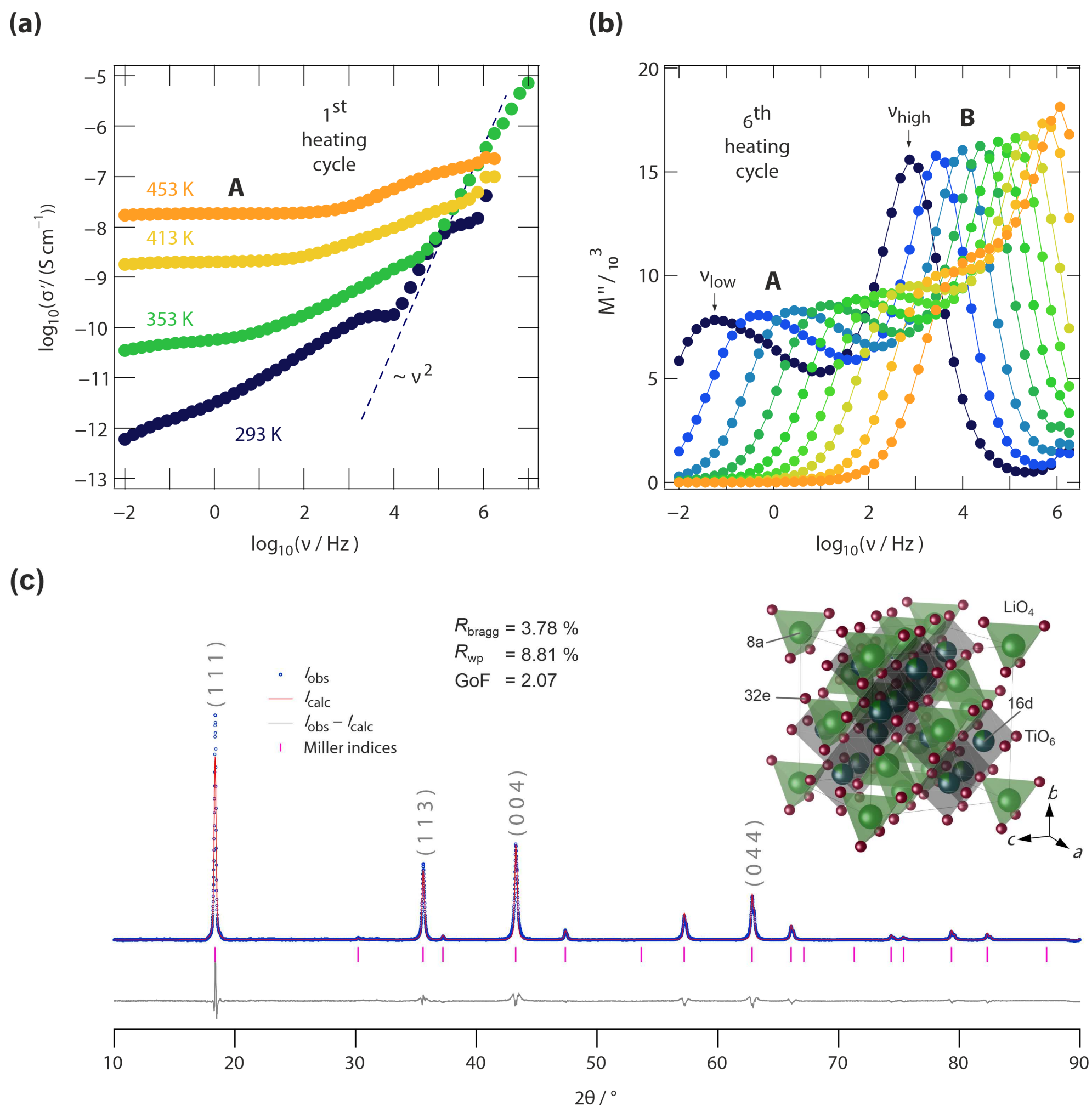

Fig. S1. (**a**) Conductivity isotherms recorded during the first heating cycle. (**b**) Imaginary part of the modulus as a function of the frequency recorded during the 6th heating cycle. The relaxation frequencies are labelled and analysed in an Arrhenius representation in Fig. 1D. (**c**) X-ray powder diffraction pattern of $Li_4Ti_5O_{12}$.

X-ray powder diffraction. The X-ray powder diffraction (XRPD) pattern of $Li_4Ti_5O_{12}$ (fig. S1c) was analyzed via the Rietveld refinement method using a single crystal reference pattern (ICSD: 160655). By using a single reference pattern, the measured XRPD pattern of our sample was well refined by the Rietveld method after subtraction of the background. However, we have to mention that one possible impurity phase, β-Li_2TiO_3 (ICSD: 57017), might be present but is hard to distinguish from LTO by XRPD as its pseudo-cubic structure yields almost identical reflexes to LTO. ^{6}Li NMR line shape measurements under MAS conditions and using the same sample did show the presence of a negligible amount of β-Li_2TiO_3 (< 3%); thus, we excluded it from our Rietveld refinement (*17*). Also, others found this phase in small amounts as an impurity in $Li_4Ti_5O_{12}$.

Site-specific ^{6}Li (MAS) NMR spin-lattice relaxation rates. We measured the spin-lattice relaxation rates $(1/T_1 \equiv R_1)$ at a Larmor frequency of 193 MHz under MAS conditions (25 kHz spinning speed) at room temperature to extract site-specific ^{6}Li NMR spin-lattice relaxation rates for spins located at the 8*a* and 16*d* positions in the LTO300 sample (see fig. S2). Integrating the peak intensities associated with Li ions in 8*a* and 16*d* sites at delay times of up to 4000 s yielded site-specific magnetization transients, which resulted in T_1 times of 19(2) s and 137(34) s, respectively.

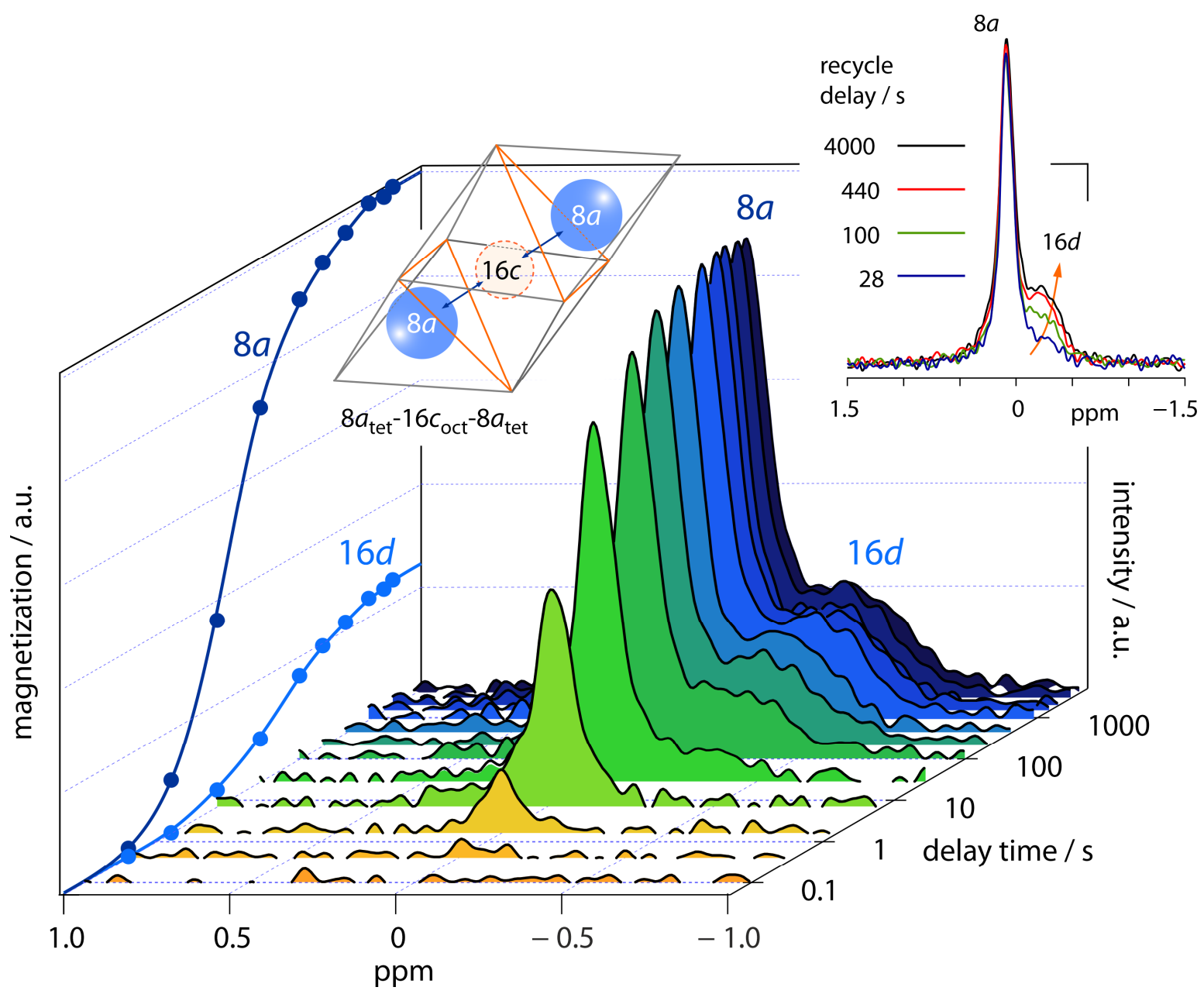

Fig. S2: Waterfall plot of the ^{6}Li MAS NMR spectra as a function of the delay time, showing how the intensity of the 8*a* and 16*d* lines evolves with increasing delay time. Separate peak integration reveals how the magnetization recovers and gives access to the site-specific T_1 relaxation times for Li ions residing on 8*a* and 16*d* (left side). The inset shows a possible jump process from 8*a* to the face-sharing octahedral site 16*c* void, causing the spin-fluctuations and, thus, longitudinal recovery.

Spin-lock ^{7}Li NMR transients. The magnetization transients recorded in the rotating frame of reference of the LTO-300 sample can be analyzed by a stretched single exponential as well as by using a stretched double exponential function (see fig. S3). A slower process contributes up to approximately 28% of the total magnetization but contributes less at higher temperatures and becomes undiscernible above 180 °C. It is worth noting that 25% of the spins are situated on the 16*d* sites, whereas the majority reside on the 8*a* positions in LTO. Hence, the distinct amplitudes may reflect the presence of two spin reservoirs in LTO. The decreasing contribution could be influenced by an increasing rate of spin exchange between the two reservoirs. Up to this temperature, we show both relaxation rates in fig. S3b, including the stretching exponents obtained by parametrizing the overall magnetization transients ei-

ther by single exponential (lines) or double exponential (circles) functions. A single exponential parameterization of the transients is biased by the large contribution of the faster process and yields almost identical relaxation rates.

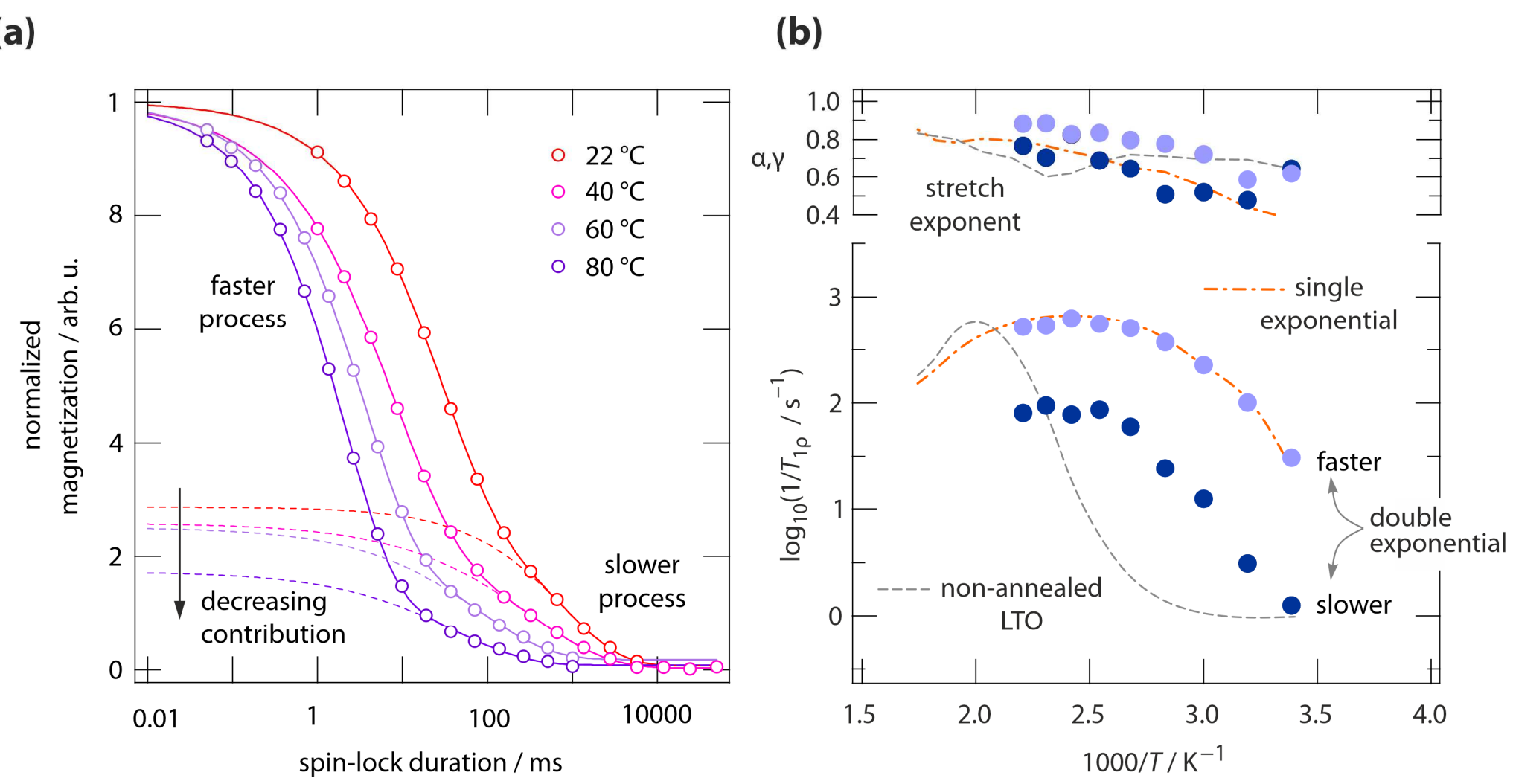

Fig. S3: (**a**) Selected ^{7}Li NMR spin-lock magnetization transients showing the contributions of a faster and a slower process to the overall observed magnetization recovery during the $T_{1\rho}$ NMR experiments at the indicated temperatures (left). Note how the contribution of the slower process decreases with increasing temperatures. At room temperature, the slow process accounts for approximately 28% of the total magnetization, which might be associated with Li on the 16*d* site. (**b**) Arrhenius plot showing the relaxation rate $1/T_{1\rho}$ and the stretching exponents used for analyzing the shape of the magnetization curves (top). If analyzed by double stretched exponentials, the two processes show a very similar behavior and are no longer separable above 180 °C. The fast process resembles the Arrhenius behavior seen when using single stretched exponential functions (green lines, see also Fig. 4A) to extract relaxation rates from the magnetization transients (see b). The rates of LTO105 are shown for comparison (grey dashed line).

Experimental

S1: The pre-exponential factor in conductivity spectroscopy.

The conductivity of a material in general depends on the charge of the mobile charge carriers, q, their numeric charge carrier density, *N*, and their electric mobility, μ, $\sigma \equiv \sigma_{DC} = Nq \cdot \mu$. The electric mobility is typically thermally activated and related to the Diffusivity via $\mu = D \cdot q / (k_B T)$ whereas the diffusivity can be traced back to the ionic hopping rate. For a 3-dimensional random walk, the diffusivity is given by $D = \nu_h \cdot a^2 / 6$ where *a* is the average jump-distance of the ion. This leads to $\sigma_{DC} T = a^2 N q^2 \nu_h / (6 k_B T)$ and links the numeric charge carrier density and the hopping rate to the conductivity. Typically, the hopping rates are thermally activated $\nu_h = \nu_0 \exp(-E_h / (k_B T))$ but also the numeric charge carrier density involved in this process may be thermally activated $N(T) = N_0 \exp(-E_N / (k_B T))$. Taking these two effects into account the thermal dependence of the specific ionic conductivity may be expressed as

$$\sigma_{DC} T = ((q^2 N_0 \nu_0) / k_B) \exp\left(-\frac{E_c + E_h}{k_B T}\right) = \sigma_0 \exp\left(-\frac{E_a}{k_B T}\right)$$

From this we can derive two important relations: (*i*) if the charge carrier density participating in a conduction process increases, the pre-exponential factor σ_0 linearly increases and (*ii*) if the charge carrier density participating in a conduction process is thermally activated, i.e. $E_h \neq 0$, the conductivity will have a higher activation energy E_a than the hopping rates E_h. For the short-range conduction mechanism (see Fig. 1) we see that the pre-exponential factor increases during in-situ annealing and matches

that of the ex-situ annealed sample. Furthermore, the charge carrier density is thermally activated judged by the difference between E_a and E_h (see Fig. 1D). The long-range conduction process shows neither of these features, *i. e.*, σ_0 remains constant and the activation energies for conduction and hopping are equal.

S2: NMR $T_{1\rho}$ maximum conditions and fitting routine

Extraction of relaxation times from magnetization transients. Magnetization transients were recorded by applying a spin-lock pulse of variable duration (t_d) after a 90° pulse and recording the following free induction decay (FID). Integrating the FID provides the magnetization $M(t_d)$ at for each spin-lock duration. A detailed description of the techniques can be found in Kuhn et al. (*56*) and therein. These transients were parameterized by using either one or two stretched exponential functions in the form of

$$M(t_d)=\left\{M_1\exp\left[-\left(t_d/T_{1\rho,1}\right)^{\alpha}\right]+M_2\right\}\exp\left[-\left(t_d/T_{1\rho,2}\right)^{\gamma}\right]$$

Here M_0, M_1 and M_2 are the residual magnetization, and the magnetization amplitudes associated with the first and the second exponential decay whereas $T_{1\rho,1}$ and $T_{1\rho,2}$ are the respective spin-lattice relaxation times. The stretching factors α and γ were restrained to $0<(\alpha,\gamma)\leq 1$.

Nuclear magnetic resonance relaxation rate measurements in the rotating frame of reference. Diffusion-induced NMR relaxation rates are proportional to the spectral density function $J(\omega)$ given by the Fourier transform of the motional autocorrelation function that represents the spin fluctuations perceived by the analyzed nucleus and is assumed to show exponential decay (*16,57*). By using a spin-lock pulse of variable duration, we can probe the spectral density function $J(\omega_{\text{lock}})$ at the locking frequency ω_{lock} instead of the orders of magnitude higher Larmor frequency $\omega_L/2\pi = 116\,\text{MHz}$. Based on the BPP (Bloembergen, Purcell, Pound) model (39) introduced for isotropic (3D) uncorrelated motion, the spin-lock relaxation rates can be expressed by

$$R_{1\rho} \propto J^{(0)}(2\omega_{\text{lock}})+10J^{(1)}(\omega_L)+J^{(2)}(2\omega_L) \propto \frac{\tau_c}{1+(2\omega_{\text{lock}}\tau_c)^{\beta}}+\frac{5}{3}\frac{\tau_c}{1+(\omega_L\tau_c)^{\beta}}+\frac{2}{3}\frac{\tau_c}{1+(2\omega_L\tau_c)^{\beta}}$$

The exponent β typically adopts values in the range of $1<\beta\leq 2$ and the inverse correlation time follows an Arrhenius-type temperature dependence as in $\tau_c^{-1}=\tau_{c,0}^{-1}\exp(-E_h/(k_B T))$. This approach is based on the BPP model and is used to parameterize the relaxation-rate peaks. Typically, the relaxation rates increase with temperature until the inverse correlation time is almost equal to ω_{lock}, after which the rates decrease. Hence, a maximum in the rates is located at that temperature at which the jump-rate of the ions, which is assumed to equal τ_c^{-1} is in the same order as ω_{lock}.

^{7}Li NMR Line shape and motional averaging. At low temperatures, the ^{7}Li NMR powder spectrum is dipolarly broadened and consists of a central line on top of a so-called quadrupolar powder pattern (see Fig. 3 F-G). With increasing temperature, the central line narrows and the quadrupolar signal undergoes motional averaging and loses intensity. The lines of LTO-105 and LTO-300 are qualitatively similar, but the motional averaging due to increased Li jump rates starts at much lower temperatures in the latter sample. Sufficiently high Li jump rates usually average these dipolar coupling, and the line narrows with increasing temperature when the jump rate reaches value in the order of the rigid lattice linewidth. In the LTO-105 sample, such motional narrowing (MN) starts at about 140 °C when the Li jump rate reaches approximately 5 kHz (see Fig. 6C). Above 260 °C, dipolar couplings are fully averaged, and the residual line width is largely determined by the inhomogeneity of the external magnetic

field B_0 of the NMR magnet. The LTO-300 sample shows a much narrower central line at 40 °C compared to the pristine sample. The jump rates in this sample are already larger than 5 kHz, and the line width is already affected by motional averaging.

S3: Converting jump rates from NMR into diffusion coefficients and final conductivities

Conversion of D_{NMR} to conductivities. The rate peaks in NMR relaxation rates give access to correlation times which are related to a diffusion coefficient via $D_{\mathrm{NMR}} = \tau_{\mathrm{c}}^{-1}\alpha^2/6$ where the mean jump distance, 1.8 x 10^{-10} m^{-1}, is given by α. Hence, using a locking frequency of 25 kHz, each maximum of the $R_{1\rho}(\equiv 1/T_{1\rho})$ rates indicates that the probed jump process is characterized by a diffusion coefficient D_{NMR} of 1.6965×10^{-15} m^2/s. The conductivity, based on this diffusion coefficient derived from NMR, can then be estimated at the temperature at which the peak is located

$$\sigma_{\mathrm{NMR}} = D_{\mathrm{NMR}} N a^2 / (k_{\mathrm{B}} T) = a^2 N q^2 \tau_{\mathrm{c}}^{-1} / (6 k_{\mathrm{B}} T)$$

Here, we assume that all Li ions reside on the 8a site. The respective numeric charge carrier density is calculated on the basis of the crystal structure and amounts to 1.37×10^{28} ions m^{-3}.

Data S1. Excel workbook (Data S1.xlsx) containing the raw numerical data underlying the X-ray diffraction and impedance spectroscopy measurements (LTO-105 and LTO-300) presented in this study. Separate worksheets provide the X-ray diffraction data (2θ and intensity) and the impedance spectroscopy data used in the analyses.

REFERENCES

1. D. Larcher, J. M. Tarascon, Towards greener and more sustainable batteries for electrical energy storage. *Nat. Chem.* **7**, 19–29 (2015).

2. R. Schmuch, R. Wagner, G. Hörpel, T. Placke, M. Winter, Performance and cost of materials for lithium-based rechargeable automotive batteries. *Nat. Energy* **3**, 267–278 (2018).

3. P. Albertus, S. Babinec, S. Litzelman, A. Newman, Status and challenges in enabling the lithium metal electrode for high-energy and low-cost rechargeable batteries. *Nat. Energy* **3**, 16–21 (2018).

4. A. A. Kebede, T. Kalogiannis, J. Van Mierlo, M. Berecibar, A comprehensive review of stationary energy storage devices for large scale renewable energy sources grid integration. *Renew. Sustain. Energy Rev.* **159**, 112213 (2022).

5. B. Dunn, H. Kamath, J.-M. Tarascon, Electrical energy storage for the grid: A battery of choices. *Science* **334**, 928–935 (2011).

6. M. Li, J. Lu, Z. Chen, K. Amine, 30 years of lithium-ion batteries. *Adv. Mater.* **30**, 1800561 (2018).

7. N. Nitta, F. Wu, J. T. Lee, G. Yushin, Li-ion battery materials: present and future. *Mater. Today* **18**, 252–264 (2015).

8. J. Li, Z.-F. Ma, Past and present of $LiFePO_4$: from fundamental research to industrial applications. *Chem* **5**, 3–6 (2019).

9. L.-X. Yuan, Z.-H. Wang, W.-X. Zhang, X.-L. Hu, J.-T. Chen, Y.-H. Huang, J. B. Goodenough, Development and challenges of $LiFePO_4$ cathode material for lithium-ion batteries. *Energ. Environ. Sci.* **4**, 269–284 (2011).

10. N. Ravet, Y. Chouinard, J. F. Magnan, S. Besner, M. Gauthier, M. Armand, Electroactivity of natural and synthetic triphylite. *J. Power Sources* **97-98**, 503–507 (2001).

11. H. Zhang, Y. Yang, H. Xu, L. Wang, X. Lu, X. He, $Li_4Ti_5O_{12}$ spinel anode: Fundamentals and advances in rechargeable batteries. *InfoMat* **4**, e12228 (2022).

12. M. M. Thackeray, K. Amine, $Li_4Ti_5O_{12}$ spinel anodes. *Nat. Energy* **6**, 683–683 (2021).

13. K. M. Colbow, J. R. Dahn, R. R. Haering, Structure and electrochemistry of the spinel oxides $LiTi_2O_4$ and $Li_{4/3}Ti_{5/3}O_4$. *J. Power Sources* **26**, 397–402 (1989).

14. W. Zhang, D.-H. Seo, T. Chen, L. Wu, M. Topsakal, Y. Zhu, D. Lu, G. Ceder, F. Wang, Kinetic pathways of ionic transport in fast-charging lithium titanate. *Science* **367**, 1030–1034 (2020).

15. J. Ma, C. Wang, S. Wroblewski, Kinetic characteristics of mixed conductive electrodes for lithium ion batteries. *J. Power Sources* **164**, 849–856 (2007).

16. W. Schmidt, P. Bottke, M. Sternad, P. Gollob, V. Hennige, M. Wilkening, Small change—great effect: steep increase of Li ion dynamics in $Li_4Ti_5O_{12}$ at the early stages of chemical Li insertion. *Chem. Mater.* **27**, 1740–1750 (2015).

17. W. Schmidt, M. Wilkening, Discriminating the Mobile Ions from the Immobile Ones in $Li_{4+x}Ti_5O_{12}$: ^6Li NMR Reveals the Main Li^+ Diffusion Pathway and Proposes a Refined Lithiation Mechanism. *J. Phys. Chem. C* **120**, 11372–11381 (2016).

18. Y. Shi, D. Zhang, C. Chang, K. Huang, R. Holze, Enhanced electrochemical performance of oxygen-deficient $Li_4Ti_5O_{12–}x$ anode material induced by graphene oxide. *J. Alloys Compd.* **639**, 274–279 (2015).

19. X. Chen, X. Guan, L. Li, G. Li, Defective mesoporous $Li_4Ti_5O_{12–}y$: An advanced anode material with anomalous capacity and cycling stability at a high rate of 20C. *J. Power Sources* **210**, 297–302 (2012).

20. C. Yang, S. Cheng, X. Yao, G. Nian, Q. Liu, Z. Suo, Ionotronic luminescent fibers, Fabrics, and other configurations. *Adv. Mater.* **32**, e2005545 (2020).

21. C. Yang, Z. Suo, Hydrogel ionotronics. *Nat. Rev. Mater.* **3**, 125–142 (2018).

22. J. C. Gonzalez-Rosillo, M. Balaish, Z. D. Hood, N. Nadkarni, D. Fraggedakis, K. J. Kim, K. M. Mullin, R. Pfenninger, M. Z. Bazant, J. L. M. Rupp, Lithium-battery anode gains additional functionality for neuromorphic computing through metal-insulator phase separation. *Adv. Mater.* **32**, e1907465 (2020).

23. B. Sreenarayanan, C.-H. Huang, R. Shimizu, S. Suwito, S. Wang, Y. B. Zheng, C.-Y. Chang, B. Han, B. Sahoo, Y. Zhang, D. P. Meganathan, K. Nomura, B. J. Hwang, W.-N. Su, Z. Jahed, M. Zhang, E. E. Fullerton, Y. S. Meng, Resistive switching of spinel $Li_4Ti_5O_{12}$ lithium-ion battery material for neuromorphic computing. *ACS Appl. Eng. Mater.* **3**, 2624–2637 (2025).

24. A. K. Jonscher, The 'universal' dielectric response. *Nature* **267**, 673–679 (1977).

25. T. Scheiber, B. Gadermaier, K. Hogrefe, R. Zettl, H. M. R. Wilkening, Thermoresistant defects preserve fast ion conduction in $LiBH_4$–ZrO_2 conductor-onsulator nanocomposites. *Chem. Mater.* **35**, 10504–10514 (2023).

26. J. T. S. Irvine, D. C. Sinclair, A. R. West, Electroceramics: characterization by impedance spectroscopy. *Adv. Mater.* **2**, 132–138 (1990).

27. B. Roling, A. Happe, K. Funke, M. D. Ingram, Carrier concentrations and relaxation spectroscopy: New information from scaling properties of conductivity spectra in ionically conducting glasses. *Phys. Rev. Lett.* **78**, 2160–2163 (1997).

28. S. Ren, J. Liu, D. Wang, J. Zhang, X. Ma, M. Knapp, L. Liu, H. Ehrenberg, Dielectric relaxation behavior induced by lithium migration in $Li_4Ti_5O_{12}$ spinel. *J. Alloys Compd.* **793**, 678–685 (2019).

29. B. Ziebarth, M. Klinsmann, T. Eckl, C. Elsässer, Lithium diffusion in the spinel phase $Li_4Ti_5O_{12}$ and in the rocksalt phase $Li_7Ti_5O_{12}$ of lithium titanate from first principles. *Phys. Rev. B* **89**, 174301 (2014).

30. C. D. Jeppe, M. Philipp, R. Bernhard, L. S. David, Fundamental questions relating to ion conduction in disordered solids. *Rep. Prog. Phys.* **72**, 046501 (2009).

31. I. M. Hodge, K. L. Ngai, C. T. Moynihan, Comments on the electric modulus function. *J. Non Cryst. Solids* **351**, 104–115 (2005).

32. Y. Gao, Z. Wang, L. Chen, Stability of spinel $Li_4Ti_5O_{12}$ in air. *J. Power Sources* **245**, 684–690 (2014).

33. F. A. Kröger, H. J. Vink, Relations between the concentrations of imperfections in crystalline solids. *Solid State Phys.* **3**, 307–435 (1956).

34. S. Zhang, W. Li, Z. Jin, J. Yang, J. Zhang, Z. Du, Z. Zhang, Study on ESR and inter-related properties of vacuum-dehydrated nanotubed titanic acid. *J. Solid State Chem.* **177**, 1365–1371 (2004).

35. H. Zhang, Y. Jiang, Z. Qi, X. Zhong, Y. Yu, Sulfur doped ultra-thin anatase TiO_2 nanosheets/graphene nanocomposite for high-performance pseudocapacitive sodium storage. *Energy Storage Mater.* **12**, 37–43 (2018).

36. E. Serwicka, M. W. Schlierkamp, R. N. Schindler, Localization of conduction band electrons in polycrystalline TiO_2 studied by ESR. *Z. Naturforsch. A* **36**, 226–232 (1981).

37. Y. Li, D.-S. Hwang, N. H. Lee, S.-J. Kim, Synthesis and characterization of carbon-doped titania as an artificial solar light sensitive photocatalyst. *Chem. Phys. Lett.* **404**, 25–29 (2005).

38. B. Qiu, M. Zhang, L. Wu, J. Wang, Y. Xia, D. Qian, H. Liu, S. Hy, Y. Chen, K. An, Y. Zhu, Z. Liu, Y. S. Meng, Gas–solid interfacial modification of oxygen activity in layered oxide cathodes for lithium-ion batteries. *Nat. Commun.* **7**, 12108 (2016).

39. N. Bloembergen, E. M. Purcell, R. V. Pound, Relaxation effects in nuclear magnetic resonance absorption. *Phys. Rev.* **73**, 679–712 (1948).

40. M. Wilkening, R. Amade, W. Iwaniak, P. Heitjans, Ultraslow Li diffusion in spinel-type structured $Li_4Ti_5O_{12}$—A comparison of results from solid state NMR and impedance spectroscopy. *Phys. Chem. Chem. Phys.* **9**, 1239–1246 (2007).

41. W. Schmidt, M. Wilkening, Diffusion-induced ^{7}Li NMR spin-lattice relaxation of fully lithiated, mixed-conducting $Li_7Ti_5O_{12}$. *Solid State Ion.* **287**, 77–82 (2016).

42. H. Hain, M. Scheuermann, R. Heinzmann, L. Wünsche, H. Hahn, S. Indris, Study of local structure and Li dynamics in $Li_{4+}xTi_5O_{12}$ ($0 \leq x \leq 5$) using ^{6}Li and ^{7}Li NMR spectroscopy. *Solid State Nucl. Magn. Reson.* **42**, 9–16 (2012).

43. K. T. Fehr, M. Holzapfel, A. Laumann, E. Schmidbauer, DC and AC conductivity of $Li_{4/3}Ti_{5/3}O_4$ spinel. *Solid State Ion.* **181**, 1111–1118 (2010).

44. M. Tatsumisago, C. A. Angell, S. W. Martin, A new problem in the correlation of nuclear-spin relaxation and ionic conductivity in superionic glasses. *J. Chem. Phys.* **97**, 6968–6974 (1992).

45. M. F. Graf, H. Tempel, S. S. Köcher, R. Schierholz, C. Scheurer, H. Kungl, R.-A. Eichel, J. Granwehr, Observing different modes of mobility in lithium titanate spinel by nuclear magnetic resonance. *RSC Adv.* **7**, 25276–25284 (2017).

46. B. Munro, M. Schrader, P. Heitjans, A.C. impedance studies of glassy and crystalline lithium aluminosilicate compounds. *Berichte der Bunsengesellschaft für Physikalische*. *Chem* **96**, 1718–1723 (1992).

47. A. Laumann, H. Boysen, M. Bremholm, K. T. Fehr, M. Hoelzel, M. Holzapfel, Lithium migration at high temperatures in $Li_4Ti_5O_{12}$ studied by neutron diffraction. *Chem. Mater.* **23**, 2753–2759 (2011).

48. H. H. Heenen, C. Scheurer, K. Reuter, Implications of occupational disorder on ion mobility in $Li_4Ti_5O_{12}$ battery materials. *Nano Lett.* **17**, 3884–3888 (2017).

49. S. Ganapathy, A. Vasileiadis, J. R. Heringa, M. Wagemaker, The fine line between a two-phase and solid-solution phase transformation and highly mobile phase interfaces in spinel $Li_{4+}xTi_5O_{12}$. *Adv. Energy Mater.* **7**, 1601781 (2017).

50. J. Zhu, J. Chen, H. Xu, S. Sun, Y. Xu, M. Zhou, X. Gao, Z. Sun, Plasma-introduced oxygen defects confined in $Li_4Ti_5O_{12}$ nanosheets for boosting lithium-ion diffusion. *ACS Appl. Mater. Interfaces* **11**, 17384–17392 (2019).

51. P. P. M. Schleker, C. Grosu, M. Paulus, P. Jakes, R. Schlögl, R.-A. Eichel, C. Scheurer, J. Granwehr, Electrolyte contact changes nano-$Li_4Ti_5O_{12}$ bulk properties via surface polarons. *Commun. Chem.* **6**, 113 (2023).

52. E. Fukushima, *Experimental Pulse NMR: A Nuts and Bolts Approach*. (CRC Press, ed. 1st, 1981).

53. V. Epp, Ö. Gün, H. J. Deiseroth, M. Wilkening, Long-range Li^+ dynamics in the lithium argyrodite Li_7PSe_6 as probed by rotating-frame spin–lattice relaxation NMR. *Phys. Chem. Chem. Phys.* **15**, 7123–7132 (2013).

54. C. P. Slichter, D. Ailion, Low-field relaxation and the study of ultraslow atomic motions by magnetic resonance. *Phys. Rev.* **135**, A1099–A1110 (1964).

55. D. Ailion, C. P. Slichter, Study of ultraslow atomic motions by magnetic resonance. *Phys. Rev. Lett.* **12**, 168–171 (1964).

56. A. Kuhn, M. Kunze, P. Sreeraj, H. D. Wiemhöfer, V. Thangadurai, M. Wilkening, P. Heitjans, NMR relaxometry as a versatile tool to study Li ion dynamics in potential battery materials. *Solid State Nucl. Magn. Reson.* **42**, 2–8 (2012).

57. A. Kuhn, S. Narayanan, L. Spencer, G. Goward, V. Thangadurai, M. Wilkening, Li self-diffusion in garnet-type $Li_7La_3Zr_2O_{12}$ as probed directly by diffusion-induced ^7Li spin-lattice relaxation NMR spectroscopy. *Phys. Rev. B* **83**, 094302 (2011).
